# Glyphosate as a Food Contaminant: Main Sources, Detection Levels, and Implications for Human and Public Health

**DOI:** 10.3390/foods13111697

**Published:** 2024-05-28

**Authors:** Juliana Maria Bitencourt de Morais Valentim, Carolina Coradi, Natália Prudêncio Viana, Tatiane Renata Fagundes, Pâmela Lonardoni Micheletti, Shaiane Carla Gaboardi, Bruna Fadel, Luciana Pizzatti, Luciano Zanetti Pessoa Candiotto, Carolina Panis

**Affiliations:** 1 Department of Pathological Sciences, Universidade Estadual de Londrina (UEL), Londrina 86057-970, Brazil; juliana.mbm25@gmail.com; 2 Center of Health Sciences, Universidade Estadual do Oeste do Paraná (UNIOESTE), Francisco Beltrão 85605-010, Brazil; carolina_coradi@hotmail.com (C.C.); nataliaprviana@gmail.com (N.P.V.); pamela.tba@hotmail.com (P.L.M.); shaiane_carla@hotmail.com (S.C.G.); lucianocandiotto@yahoo.com.br (L.Z.P.C.); 3 Department of Biological Sciences, Universidade Estadual do Norte do Paraná (UENP), Bandeirantes 86360-000, Brazil; tatiane.fagundes@hotmail.com; 4 Instituto Federal Catarinense, Blumenau 89070-270, Brazil; 5 Laboratório de Biologia Molecular e Proteômica do Sangue, Instituto de Química, Universidade Federal do Rio de Janeiro (IQ-UFRJ), Rio de Janeiro 21941-909, Brazil; brunaluisa.fadel@gmail.com (B.F.); pizzatti@gmail.com (L.P.)

**Keywords:** pesticides, food, glyphosate, risk assessment

## Abstract

Glyphosate is a broad-spectrum pesticide that has become the most widely used herbicide globally. However, concerns have risen regarding its potential health impacts due to food contamination. Studies have detected glyphosate in human blood and urine samples, indicating human exposure and its persistence in the organism. A growing body of literature has reported the health risks concerning glyphosate exposure, suggesting that the daily intake of contaminated food and water poses a public health concern. Furthermore, countries with high glyphosate usage and lenient regulations regarding food and water contamination may face more severe consequences. In this context, in this review, we examined the literature regarding food contamination by glyphosate, discussed its detection methods, and highlighted its risks to human health.

## 1. Introduction

Food production is closely linked to pesticide use, with the argument being that pesticides protect crops from diseases and pests, thereby increasing yields and food quality. However, this assertion is not widely accepted, primarily due to the risks pesticide exposure poses to humans. The presence of pesticide residues in food is considered a significant global health issue today [1]. Excessive and improper use of pesticides, especially in developing countries, can lead to environmental pollution and have long-term adverse effects on human health. Monitoring the environmental residues of pesticides and human exposure throughout the production chain is essential to enforcing legislation and ensuring food safety [2]. These chemicals are classified into more than a hundred classes, and the most used today are organochlorines (OCPs), organophosphates (OPPs), carbamates, pyrethroids, neonicophenoxy alkanes, and glyphosate-based pesticides [3]. Countries allow specific pesticides according to individual local conditions, and the type of pesticide used varies depending on the type of pest emerging during the growing season [4,5]. In this way, each country defines the maximum pesticide residue limits (MRLs) allowed in agricultural products [6,7].

Concerning food intake safety, the Joint Meeting on Pesticide Residues (JMPR), which comprises the World Health Organization (WHO) Core Assessment Group and the Food and Agriculture Organization Panel of Experts on Pesticide Residues in Food and the Environment, established food toxicological parameters, such as acceptable daily intake and acute reference dose, based on experimental data, recommending maximum pesticide residue concentrations (maximum residue levels, or MRLs) for consideration by the Codex Committee on Pesticide Residues (CCPR) [6]. Chronic exposure to pesticides causes negative effects on the endocrine, immune, and neurological systems, including kidney and liver problems, respiratory complications, and birth defects, and increases susceptibility to several human cancers, including head, neck, lung, breast, cervix, prostate, thyroid, brain, colorectal, pancreatic and lung cancer, and leukemia [1]. The MRLs adoption aims to provide a wide margin of food safety based on good agricultural practices. Despite this, they differ considerably among countries worldwide [7]. Thus, each country defines acceptable concentrations of certain pesticides in food and water and restricts or prohibits using certain pesticides due to their unacceptable effects on health or the environment.

Certain contaminants have garnered significant attention due to their widespread global use, with glyphosate being a primary focus. As the most widely used organophosphate herbicide, glyphosate is traded under various brand names worldwide, with hundreds of glyphosate-based herbicides (GBHs) in use [8,9,10]. Glyphosate residues are frequently detected in the environment, including in plants, soil, water, food products, and human fluids [11], utilizing various analytical methods [12,13,14,15,16]. Consequently, concerns within the scientific community have escalated regarding the potential environmental and human health impacts of this herbicide and its metabolites.

In this review, we analyze the literature on glyphosate as a contaminant in food, highlighting the strengths and limitations of available analytical methods for its detection. Furthermore, we discuss the potential risks this contamination poses to human health. A literature review was conducted to gather information on the presence of glyphosate in foods, its analytical detection methods, and its possible consequences on human health. The platforms employed to collect data included PubMed, Google Scholar, and Web of Science. The following keywords were used individually and in combination: glyphosate, herbicide, food, aminomethylphosphonic acid (AMPA), and glyphosate detection. Articles published on any date were included. The authors screened titles and abstracts to classify eligible articles and reviewed the full text. All pieces included were written and published in English. Animal, in vitro, cross-sectional, case-control, cohort, and ecological studies were evaluated. Sponsored publications or publications with authors linked to the herbicide industry were excluded. Publications related to analytical methodologies that did not present detection or quantification limits were also discarded.

## 2. Glyphosate Use

**Figure 1:**
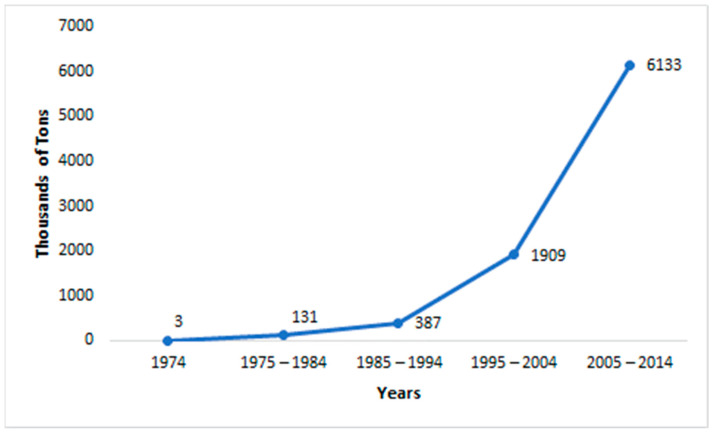
Total global glyphosate active ingredient uses by decade.

Glyphosate N-(phosphonomethyl)-glycine is an herbicide that belongs to the family of organophosphorus compounds (1 Commission Regulation (EU) 2017/269 of 16 February 2017; Official Journal of the European Union: Brussels, Belgium, 2017; L. 40/4.). It started to be sold in 1974. As of today, it is the most intensively and widely used pesticide around the world [17]. In 1974, its first year of commercialization, the global glyphosate consumption was about 3000 tons. In 1994, there were 56,000 tons; in 2014, global glyphosate usage reached the 826,000 tons mark [9]. The rise in glyphosate consumption is illustrated in Figure 1.

Since the mid-1990s, the American continent has been the world’s largest consumer of pesticides, surpassing Asia, Europe, Africa, and Oceania. Between 1990 and 2021, there was a 191% increase in pesticide use in the Americas, with an average use of approximately 1.12 million tons per year. Additionally, the American continent was also responsible for importing the largest quantity of active ingredients from other regions of the world in 2021, amounting to a value of USD 7.6 billion and 1.2 million tons of formulated products. In this scenario, three American countries are among the top five pesticide users globally: Brazil (720,000 tons), the United States (457,000 tons), and Argentina (242,000 tons), with the latter using quantities similar to China and Indonesia. Considering pesticide application per cultivated area, Brazil (10.9 kg/ha) and Argentina (5.6 kg/ha) lead, followed by Indonesia (5.3 kg/ha), Spain, and France (<5.3 kg/ha) [18].

Furthermore, the product is sold most in the countries with the highest pesticide usage globally. In Brazil, for instance, approximately 30% of the total volume of pesticides sold in the country comprises the active ingredient glyphosate and its salts [19].

Since 1995, glyphosate has been an herbicide used in genetically modified organisms (GMOs) resistant to active ingredients, such as soybeans, corn, and cotton. Bohn and Millstone [20] focused on the expansive growth of glyphosate use, highlighting Roundup Ready herbicide, with the introduction of GMO soybean (Glyphosate Tolerant Soybeans). When GT soybeans were initially commercialized in 1995, the company that created this soybean said it would reduce the necessity of herbicide application. However, the authors showed that farmers from the United States, Argentina, and Brazil have doubled their glyphosate applications per season.

About 77% of the global soybean production comes from GT soybean, and the dominant soy-producing countries of Brazil, the USA, and Argentina have a 94–100% adoption rate of ‘biotech crops’, mostly glyphosate-tolerant varieties [21]. Generally, glyphosate is applied at three stages: before planting to eliminate spontaneous plants and avoid plowing, during the crop season to control weeds, and pre-harvest as a desiccant to accelerate maturation and dry the seeds. However, it is also used to clear soil under perennial crops, in urban areas, and in gardening, replacing manual weeding.

The extensive use of glyphosate has sparked concerns among researchers, as well as health and environmental institutions worldwide [22]. One of the primary areas of concern is the high tolerance for residues of this active ingredient in drinking water and food in certain countries. Regarding drinking water, the maximum residue limit set in the European Union is established based on the precautionary principle. When compared to major agricultural commodity producers, such as Brazil and the United States, the difference can be as much as 5000 and 7000 times higher, respectively.

For instance, the disparity between the maximum residue limits set in the European Union and Brazil is not limited to drinking water. It is also evident in the allowable residue levels for the cultivation of coffee (10 times higher), sugarcane (20 times higher), and soybeans (200 times higher) [23]. Glyphosate residues in drinking water, vegetables, and processed food result from the intensive use of this active ingredient worldwide. Through a literature review, Soares et al. [24] discussed glyphosate use, toxicity, and occurrence in diverse kinds of food. The paper highlights the use in Europe, where France was the country with primary consumption in 2018 (35,000 tons), and Belgium was the country that led use intensity (1.96 kg/ha). It also discusses human toxicity and glyphosate in olive oil, honey, fruits and nuts, cereals and cereal products, vegetables, animal-derived products, baby food, water, and alcoholic beverages.

Current studies have demonstrated that the presence of glyphosate and/or its metabolite, AMPA, may compromise the quality of soil and water, as well as the health of plants, animals, and humans. Glyphosate residues in soil, surface water, and groundwater have been widely detected in areas where genetically modified organisms resistant to the active ingredient are cultivated [25].

Furthermore, glyphosate has caused direct and indirect impacts on ecosystems, leading to a reduction in biodiversity at various scales. Pest control by natural predators, insect pollination, and the functional structure of the soil are threatened by the elimination of wild plants from agricultural fields and nearby lands. Additionally, there are direct toxic effects on many species within soil microbial communities, worms, pollinators, and the plant’s defense mechanism, rendering them more susceptible to pathogens and diseases [26].

Aquatic organisms are also impacted by the presence of glyphosate in water. Costa et al. [27] assert that high doses of glyphosate, although within acceptable regulatory guidelines, can alter freshwater bacterioplankton. This alteration ultimately favors a subset of higher taxonomic units (from genus to phylum) that transiently thrive in glyphosate-contaminated environments. Lanzarin et al. [28] suggest that glyphosate-based formulations may induce neurotoxic effects in fish, leading to behavioral deficits. Furthermore, glyphosate affects amphibians, causing osmotic instability, delayed or accelerated development, reduced size at metamorphosis, malformations, stress, and even death [29].

The contamination of ecosystems also has social implications. Environmental and dietary exposure to glyphosate makes humans increasingly susceptible to potential deleterious health effects. Studies have shown that glyphosate and AMPA are present in the bodies of the population, indicating a collective health problem. Examples include the studies by Palma et al. [30] and Camiccia et al. [31], which detected glyphosate in breast milk samples from infants in the Midwest and South of Brazil, respectively, demonstrating that babies are also vulnerable, despite having developing immune systems.

Other studies have indicated the presence of glyphosate in the human body. In South America, Candiotto et al. [32] detected glyphosate-AMPA in 90% of urine samples collected from residents of a rural village in the Southern region of Brazil who were exposed to pesticide drift from the spraying of neighboring crops. Other researchers have recently detected the active ingredient in urine samples in North America [33,34], Europe [35,36], Asia [37], Oceania [38], and Africa [39]. Additionally, in Brazil, extensive contamination of drinking water has been recently documented by Panis et. al., (2022) [40], and higher cancer risk is anticipated for populations consuming contaminated water by glyphosate and other pesticides. This reaffirms that glyphosate residues are jeopardizing the human and environmental health of the entire planet and suggests a possible link between glyphosate contamination and food/water consumption.

## 3. Chemical Properties and Analytical Detection of Glyphosate

**Figure 2:**
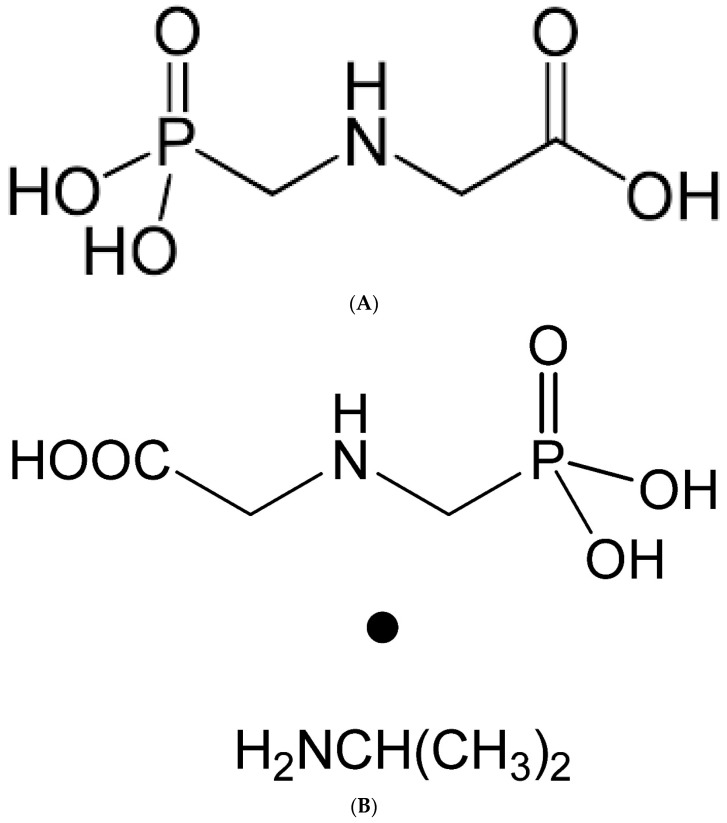
Structural formula of glyphosate (**A**) and glyphosate-isopropylammonium (**B**).

Glyphosate (Figure 2A) is a salt composed of deprotonated glyphosate acid and a cation (isopropylamine, ammonium, or sodium), has a molecular formula C3H7NO5P (m.m. = 169.05 g/mol) and, in the compound isopropylammonium salt, is added to the group (CH3)2 CHNH3 + (m.m. = 228.2 g/mol, Figure 2B). It is a crystalline, odorless, and colorless solid. Under ambient conditions, both glyphosate and its salts are crystalline solids, very soluble in water (>353 mg/mL (pH 7; 20 °C)) and practically insoluble in common organic solvents, such as acetone and ethyl acetate [41].

Glyphosate melts at ~235 °C and has a bulk density of 1.655 g/mL (20 °C). It is stable for hydrolysis from pH 3 to pH 9 and relatively stable for photodegradation [42]. It decomposes on heating, producing toxic vapors, including nitrogen and phosphorus oxides [43]. The pK values for glyphosate are pK1 = 2.72; pK2 = 5.63; pK3 = 10.2 [41]. Its conversion factor, assuming normal temperature (25 °C) and pressure (101 kPa), is mg/m^3^ = 6.92 × ppm [44]. All properties are summarized in Table 1.

Glyphosate is a non-selective systemic crop protection product widely used in agriculture to control the growth of weeds. It is considered one of the most popular herbicides in the world due to its effectiveness. It is effective against over 100 broadleaf weeds and grasses and more than 60 perennial weeds [46]. It can be found in soil, air, surface water, and groundwater [47]. The term glyphosate indicates both the acid and its salts, as it is recognized that they are biologically equivalent [48]. The degradation of glyphosate in the soil is very rapid and carried out by a wide variety of microorganisms, producing AMPA as the primary metabolite and sarcosine as an intermediate metabolite in the alternative route [49,50]. According to the National Health Surveillance Agency [51], in Brazil, glyphosate is the active component most used in Brazilian plantations, resulting in, in 2017, a total of 173,150.75 tons of the active ingredient alone, which also comprises its salts, and are classified as ready-to-use products.

With the wide variety of pesticides used in crop production, there is a need for multi-residue detection methods. These methods usually involve extraction/partition, sample cleaning, and instrumental analysis to detect multiple pesticide classes in a single procedure [52]. Commonly employed extraction solvents include acetonitrile (QuEChERS), ethyl acetate (SweEt), and acetone (NL). Liquid chromatography coupled with mass spectrometry (LC-MS) and gas chromatography coupled with mass spectrometry (GC-MS) are primarily employed for pesticide analysis owing to their high sensitivity and selectivity. To enhance the effectiveness of these methods, extensive sample preparation and optimization protocols are employed to ensure adequate compound separation and minimize matrix effects [52,53,54].

Glyphosate, an extensively utilized systemic herbicide in agriculture, presents analytical complexities due to its chemical characteristics, such as its high polarity and propensity to form metal complexes. Glyphosate, and its metabolite aminomethylphosphoric acid (AMPA), lack volatility and chromophores, making direct analysis difficult using GC or LC techniques [54,55,56]. Consequently, various derivatization techniques have been proposed, such as pre-column derivatization with 9-fluorenylmethyl chloroformate (FMOC-Cl) or post-column derivatization with phthaldehyde (OPA) and mercaptoethanol. However, these methods are time-consuming and may lack certainty regarding derivatization success.

Alternative methodologies for direct analysis of polar compounds have emerged, including ion chromatography, graphitized carbon columns (Hypercarb), and hydrophilic interaction liquid chromatography (HILIC) [55,56]. In addition to GC-MS, GC-MS/MS, liquid chromatography with fluorescence detector (LC-FLD), and liquid chromatography with UV/Vis detector (LC-UV) methods are employed, often requiring analyte derivatization and a cleaning or pre-concentration step [54,56].

Mass spectrometry techniques, including liquid chromatography-mass spectrometry (LC-MS) and gas chromatography-mass spectrometry (GC-MS), offer precise quantification and identification of glyphosate and its metabolites in complex biological matrices. Mass spectrometry techniques play a pivotal role in detecting glyphosate in human diseases, offering high sensitivity, specificity, and accuracy for biomonitoring and risk assessment purposes. LC-MS, GC-MS, HR-MS, and MS/MS methods have been instrumental in elucidating the relationship between glyphosate exposure and various health outcomes, highlighting the importance of robust analytical methodologies in assessing human health risks associated with environmental contaminants like glyphosate [57].

LC-MS is a widely used technique for glyphosate detection in human diseases due to its high sensitivity and selectivity. The method involves the separation of analytes by liquid chromatography followed by ionization and detection by mass spectrometry. Various LC-MS methods, such as LC-MS/MS and LC-QTOF, have been employed for glyphosate quantification in urine, blood, and tissue samples with excellent sensitivity and accuracy for quantifying glyphosate at trace levels. These methods provide excellent chromatographic resolution, enabling the separation of glyphosate from matrix interferences. They require minimal sample preparation, reducing the risk of analyte loss or degradation. Additionally, they are suitable for high-throughput analysis due to fast chromatographic separations and short analysis times. Regarding limitations, LC-MS may suffer from matrix effects in complex biological samples, affecting the accuracy and reproducibility of quantification. The instrumentation and maintenance costs associated with LC-MS systems can be high, limiting accessibility for some research groups [57,58].

GC-MS is another valuable tool for glyphosate analysis, particularly in environmental and biological samples. This method involves the separation of volatile compounds by gas chromatography followed by ionization and detection using mass spectrometry. GC-MS offers excellent resolution and sensitivity for glyphosate detection in complex matrices, although sample derivatization may be required for optimal sensitivity. This method is well-suited for volatile and semi-volatile compounds like glyphosate and its derivatives. It requires standardization of sample derivatization to enhance the volatility and thermal stability of glyphosate for optimal detection. Additionally, it offers excellent sensitivity and resolution, particularly for analyzing glyphosate in environmental samples. However, it may suffer from matrix effects in complex biological matrices, requiring careful method development and validation [57,58,59]. This method is also useful for quantifying glyphosate residues in food, water, and soil samples due to its robustness and sensitivity. Sample derivatization is a limitation and is often required to enhance the volatility of glyphosate, which can increase analysis time and complexity. GC-MS may not be suitable for analyzing glyphosate in biological matrices due to matrix effects and interference from endogenous compounds.

HR-MS techniques, such as quadrupole time-of-flight (Q-TOF) and Orbitrap MS, provide enhanced mass resolution and accurate mass measurements, enabling reliable glyphosate identification and quantification in human samples such as urine and serum. These methods offer superior performance in distinguishing glyphosate from its metabolites and other interfering compounds, thereby improving analytical specificity and allowing discrimination between glyphosate and structurally similar compounds. These methods enable comprehensive screening of glyphosate residues in human samples without prior knowledge of analyte masses. They are suitable for non-targeted analysis to identify unknown glyphosate-related compounds or metabolites. They require advanced instrumentation and expertise, potentially limiting accessibility and throughput in routine analysis. The method development and optimization may require specialized expertise, particularly for non-targeted analysis approaches [58,59].

Techniques involving multiple reaction monitoring (MRM) and selected reaction monitoring (SRM) in tandem mass spectrometry (MS/MS) provide improved sensitivity and specificity for accurately quantifying glyphosate in complex biological samples. By monitoring specific precursor-product ion transitions, MS/MS enables precise quantification of glyphosate at low concentrations, even in the presence of matrix interferences. It facilitates multiplexed analysis of glyphosate and its metabolites in a single run, improving analytical efficiency [57,58,59]. It requires optimization of collision energies and fragmentation conditions to maximize sensitivity and specificity. Developing and optimizing MS/MS methods extensively may be necessary to attain the desired sensitivity and selectivity. The complexity of MS/MS data analysis and interpretation may require specialized training and expertise. Despite that, it is widely used in targeted biomonitoring studies to assess glyphosate exposure and its association with human diseases [60].

In general, each mass spectrometry method offers unique advantages and limitations for glyphosate detection. LC-MS and GC-MS are well-suited for specific sample types (biological vs. environmental) and analyte characteristics (polarity, volatility). HR-MS techniques provide superior resolution and selectivity but may require more resources and expertise. MS/MS methods offer excellent sensitivity and specificity but may necessitate method optimization and data analysis skills. The choice of method should consider factors such as sample matrix, analyte concentration, desired sensitivity, and available resources. Future studies should prioritize resolving methodological obstacles and standardizing analytical techniques to ensure precise and dependable analysis of glyphosate in human diseases [59,60].

Antibody-based immunoassays represent a cornerstone in the detection of pesticides, including glyphosate, providing sensitive and specific analytical tools for food safety and environmental monitoring. This review offers an in-depth examination of antibody-based immunoassay techniques for pesticide detection, highlighting their principles, applications, strengths, and limitations. Furthermore, a comparative analysis with mass spectrometry methods is presented, discussing their respective advantages and challenges in pesticide analysis.

Antibody-based immunoassay techniques have emerged as powerful tools for pesticide detection, offering high sensitivity, specificity, and rapid analysis capabilities. Antibody-based immunoassays rely on the specific binding interaction between antibodies and target analytes, such as pesticides. Various immunoassay formats, including enzyme-linked immunosorbent assay (ELISA), immunochromatographic assay (ICA), and surface plasmon resonance (SPR) biosensors, have been developed for pesticide detection. These techniques offer sensitive and rapid detection of pesticides in complex sample matrices, making them suitable for routine monitoring and regulatory compliance [61,62].

Antibody-based immunoassays find extensive applications in pesticide detection across diverse sample matrices, including food, water, soil, and air. They are utilized in both laboratory and field settings for screening, monitoring, and surveillance purposes. Commercially available immunoassay kits enable rapid and on-site detection of pesticides, facilitating timely decision-making in agricultural and environmental management [61].

Despite their advantages, antibody-based immunoassays face challenges such as cross-reactivity, matrix effects, and assay validation. Ongoing research efforts focus on improving assay performance through the development of novel antibodies, signal amplification strategies, and integration with emerging technologies. Furthermore, advancements in microfluidics and nanomaterials hold promise for enhancing the sensitivity, specificity, and portability of immunoassay-based pesticide detection systems [63,64].

In comparison with mass spectrometry methods, including liquid chromatography-mass spectrometry (LC-MS) and gas chromatography-mass spectrometry (GC-MS), for pesticide analysis, mass spectrometry offers unparalleled sensitivity and multi-residue analysis capabilities, it requires sophisticated instrumentation, extensive sample preparation, and skilled operators. In contrast, antibody-based immunoassays provide rapid screening and cost-effective analysis but may lack the quantitative accuracy of mass spectrometry [65]. Antibody-based immunoassay techniques play a vital role in pesticide detection, offering sensitive, specific, and rapid analytical methods for food safety and environmental monitoring. While mass spectrometry methods excel in sensitivity and multi-residue analysis, they pose challenges in terms of complexity and cost. Integration of both techniques may enhance overall detection capabilities and address the diverse needs of pesticide analysis in various applications [65,66].

ELISA-based assays have emerged as indispensable tools for pesticide detection, offering rapid, sensitive, and cost-effective analytical methods for routine screening and regulatory compliance. ELISA is a widely employed immunoassay technique for pesticide detection, based on the specific binding interaction between antibodies and target pesticides. In a typical ELISA setup, antibodies specific to the target pesticide, such as glyphosate, are immobilized onto the surface of a microplate well. The sample containing the pesticide is introduced into the well, allowing the pesticide molecules to bind to the immobilized antibodies [67]. After washing away unbound components, an enzyme-conjugated secondary antibody is added, which binds to the pesticide–antibody complex. The addition of a substrate solution triggers an enzyme-mediated colorimetric or fluorescent reaction, generating a detectable signal proportional to the pesticide concentration in the sample. Recent advancements in ELISA technology focus on enhancing assay sensitivity, specificity, and robustness through the development of novel antibodies, signal amplification strategies, and miniaturization techniques [67,68].

Despite their advantages, ELISA techniques face challenges such as matrix effects, cross-reactivity, and assay validation, necessitating ongoing research efforts to address these issues. Integration of ELISA with emerging technologies, such as microfluidics and nanomaterials, holds promise for improving analytical performance and portability in pesticide detection. With ongoing advancements, ELISA techniques will continue to play a crucial role in ensuring pesticide safety and sustainability in agricultural and environmental practices.

Immunochromatographic assays (ICAs) have emerged as rapid, sensitive, and portable methods for pesticide detection, offering practical solutions for on-site analysis in diverse environmental matrices [63]. ICAs are based on the specific binding interaction between antibodies and target analytes, such as pesticides. In a typical ICA setup, antibodies specific to the target pesticide, such as glyphosate, are immobilized as a line on a nitrocellulose membrane within a plastic test strip. The sample containing the pesticide is applied to the sample pad and flows through the strip via capillary action [63,69,70]. If the pesticide is present in the sample, it forms a complex with labeled antibodies conjugated to gold nanoparticles, producing a visible line at the detection zone. The appearance and intensity of the line indicate a positive result, with the intensity correlating with the pesticide concentration in the sample. ICAs find diverse applications in pesticide detection across various sample matrices, including food products, water sources, soil, and environmental samples [70,71]. Recent advancements in ICA technology focus on enhancing assay sensitivity, specificity, and robustness through the development of novel antibodies, signal amplification strategies, and improved detection platforms. Despite their advantages, ICAs face challenges, such as sensitivity limitations, matrix effects, and assay validation, necessitating ongoing research efforts to address these issues.

Surface plasmon resonance (SPR) biosensors offer label-free, sensitive, and real-time analytical methods for pesticide detection, making them invaluable tools in regulatory compliance, environmental monitoring, and food safety assessment. SPR biosensors exploit the phenomenon of surface plasmon resonance, which occurs when polarized light interacts with free electrons at the interface between a metal surface, typically gold or silver, and a dielectric medium, such as the sample solution [72]. In an SPR biosensor setup, antibodies specific to the target pesticide, such as glyphosate, are immobilized onto the sensor surface. When the sample containing the pesticide flows over the sensor surface, binding of the pesticide to the immobilized antibodies causes changes in the refractive index, resulting in a shift in the SPR signal. Monitoring the shift in SPR signal in real-time enables quantitative analysis of the pesticide concentration in the sample without the need for labeling or secondary reactions [72,73]. This offers rapid, sensitive, and label-free detection of pesticides, enabling real-time monitoring and quantitative analysis. SPR biosensors are utilized in both laboratory and field settings for screening, monitoring, and regulatory compliance purposes. The integration of SPR biosensors with microfluidic systems further enhances their analytical capabilities, enabling high-throughput analysis and sample multiplexing [72]. Recent advancements in SPR biosensor technology focus on enhancing assay sensitivity, specificity, and robustness through the development of novel sensor designs, surface chemistries, and signal amplification strategies. Despite their advantages, SPR biosensors face challenges, such as sample matrix effects, non-specific binding, and assay optimization, which necessitate ongoing research efforts to address. Integration of SPR biosensors with emerging technologies, such as nanomaterials and machine learning algorithms, holds promise for improving analytical performance and addressing complex sample matrices in pesticide detection [63,74].

## 4. Contaminated Food as a Source of Glyphosate Ingestion

Consumption of food and water is a significant source of chronic and ongoing glyphosate contamination. Residues of glyphosate and its metabolites pose long-term risks to human health, especially when they exceed the Maximum Residue Limit (MRL). The MRL is determined based on the consumption of milligrams of the herbicide’s product and by-product per kilogram of the individual [2].

Pesticide residues are detected in many vegetables and products of animal origin [24]. The MRL for glyphosate varies according to the current legislation in each country. In Brazil, the Food Pesticide Residue Analysis Program (PARA) released a report in 2020 regarding the detection of glyphosate and AMPA (aminomethylphosphonic acid) in samples from 2017 and 2018. The report indicated that the herbicide was detected in samples of rice, grapes, and mango, which represented 2% of the samples and are not recommended for cultivation [75]. Analysis of subsequent years is ongoing (see Supplementary Table S1).

Reports issued by EFSA (European Food Safety Authority) from 2018 to 2020 indicate residual detection of the herbicide in samples of barley, lentils, wheat, buckwheat, oats, and rye, where the LOQ varies between 0.002 and 0.01 mg/kg [76] (Supplementary Table S2).

In the same report, products of animal origin were also evaluated and found to contain glyphosate, honey being one example of this. Still at the European level, in 2019, a report made available by the European Union [76] (Supplementary Table S3) pointed to detection above the MRL in samples of grains, such as beans, buckwheat, and millet, where 7.7% of samples exceeded the limit considered non-toxic for human consumption. In addition to these results, other food items also showed traces of the active ingredient in cabbage, spinach, peaches, honey, and their derivatives.

In North America, the FDA (Food and Drug Administration), a regulatory body in the United States of America, in 2018 and 2019 (Supplementary Table S4) detected glyphosate in soybeans and corn in 61.12% and 57.5% of samples, respectively [77,78]. In Canada, the CFIA (Canadian Food Inspection Agency, (Supplementary Table S5)) pointed out the presence of glyphosate in several fresh, processed foods and beverages [79].

Although the MRL (Maximum Residue Limit) is variable, it is important to emphasize that its determination is often quantified based on the results of toxicity tests on animals and other bioindicator organisms [80,81]. Therefore, the MRL, whether in foods of plant or animal origin, does not necessarily mean an amount of residue safe for ingestion. Due to the wide use of the herbicide in cultivars, more and more studies have been observed that show alarming results in the detection of glyphosate and/or its metabolites in water, food, and animal and human waste [82,83,84].

Several studies have reported the presence of glyphosate in foods, aiming to evaluate possible irregularities and risks to human health in countries that consume significant quantities of foods containing this particular pesticide. To this end, several analytical techniques have been employed. Liquid (LC) or gas (GC) chromatography coupled with mass spectrometry (MS) is highlighted as a bioanalytical method widely applied to glyphosate detection. This technique is used for separating and purifying such substances through a two-phase system combined with MS, which identifies and quantifies compounds based on the molecules’ mass–charge relationship (m/z) [85]. LC-MS/MS (Liquid chromatography coupled with triple quadrupole mass spectrometry) offers several advantages in this context, such as high sensitivity and selectivity and detection of compounds even at low concentrations. The technique also allows the identification of more stable glyphosate degradation products, such as AMPA. The main limitation of its use is the high cost per sample, which limits its use as a routine method in sample screening. In Table 2, we provide detailed information about studies reporting food contamination by glyphosate, including its levels and the analytical method employed.

**Table 2:** Studies reporting glyphosate detection in food and water samples.

Reference	Analytical Method	Sample	Residue	Considerations
Otmar Zoller et al., 2018 [86].	LC-MS/MS	Food	Glyphosate, AMPA (aminomethylphosphonic acid)	The LOQ for solid samples was 0.001 mg/kg for glyphosate and 0.0025 mg/kg for AMPA. For liquid samples the LOQ was 0.0005 mg/kg for glyphosate and ranged from 0.0005 to 0.001 mg/kg for AMPA.
Sara Savini et al., 2019 [87].	UHPLC-ESI-MS/MS	Processed fruits and vegetables	Glyphosate, AMPA (aminomethylphosphonic acid)	Glyphosate was detected in 15 samples (18%) with concentrations ranging from 0.003 to 0.01 mg/kg. Only two samples of canned vegetables surpassed the MRL of 0.1 mg/kg, measuring 0.3 and 0.2 mg/kg, respectively. AMPA residues were found in two samples (orange juice and canned vegetable), both at the LOQ of 0.003 mg/kg.
Noëmie El Agrebi et al., 2020 [88].	HPLC-ESI-MS/MS	Honey	Glyphosate, AMPA (aminomethylphosphonic acid)	In bee bread, 81.5% of the samples showed a residue concentration higher than the LOQ and 9.9% showed a concentration below the LOQ, indicating detection without quantification. In beeswax 26% of samples exceeded the LOQ versus 81.5% exceeded the LOQ.
Cintia F.R. Mendonça et al., 2020 [89].	HPLC	Water	Glyphosate, AMPA (aminomethylphosphonic acid)	The water samples exhibited glyphosate concentrations ranging from 0.31 to 1.65 μg/L. AMPA levels varied between 0.50 and 1.40 μg/L. Glyphosate was detected in 19.3% of samples and quantified in 17.7%, while AMPA was detected in 21.8% of samples and quantified in 1.6%.
Maria C. Fontanella et al., 2022 [90].	HPLC-ICP-MS/MS	White (WR) and brown (BR) rice	Glyphosate	The LOD was 0.0027 mg/kg for WR and 0.0136 mg/kg for BR, while the LOQ was 0.0092 mg/kg for WR and 0.0456 mg/kg for BR.
Ana P.F de Souza et al., 2021 [91].	HPLC	Honey	Glyphosate, AMPA (aminomethylphosphonic acid)	Six samples showed glyphosate levels above the EU maximum residue limit of 0.05 µg/g, and one sample showed AMPA at 0.10 µg/g.
Maria C. Arregui et al., 2004 [92].	HPLC	Soy	Glyphosate	In soybean leaves and stems, glyphosate residues range from 1.9 to 4.4 mg/kg, while in grains they range from 0.1 to 1.8 mg/kg.
Abukari Wumbei et al., 2019 [93].	LC-MS/MS	Yam	Glyphosate	Of the 68 samples examined, glyphosate was detected in 14, albeit at levels below the LOQ.
Nicoleta Suciú et al., 2023 [94].	UHPLC-ESI-MS/MS	Water	Glyphosate, AMPA (aminomethylphosphonic acid)	Glyphosate was found in 40% of groundwater samples. AMPA was detected in 55% of the samples, of which 56% presented values above the groundwater equilibrium level.
Angela Santilio et al., 2019 [95].	LC/MS/MS	Corn and rice	Glyphosate	Average recoveries for both matrices ranged between 70 and 105% at three fortification levels, including LOQ. The LOD was determined to be 0.002 mg/kg for rice and 0.004 mg/kg for corn. The LOQ was set at 0.01 mg/kg for both corn and rice.
Nádia R. de Souza, 2018 [96].	HPLC	Baby foods	Glyphosate, AMPA (aminomethylphosphonic acid)	Among samples containing levels above the LOQ, glyphosate residues ranged from 0.03 mg/kg to 1.08 mg/kg, while AMPA residues ranged from 0.02 mg/kg to 0.17 mg/kg.
Xiu Y. Jing et al., 2021 [97].	HPLC	Medlar (leaves, soil, groundwater and honey)	Glyphosate	A total of 76 samples were analyzed and residues of four (36.7%) compounds were detected in the samples. Glyphosate was the predominant pesticide detected in soil samples (ranging from 0.21 to 1.3 mg/kg).
Mārtiņš Jansons et al., 2018 [98].	LC-MS/MS	Beer	Glyphosate	The glyphosate concentration in beer was below LOD, ranging from 0.2 μg/kg to 150 μg/kg. Beers without country-of-origin indication on the label exhibited a significantly higher glyphosate content (*p* < 0.01). The average concentration was 1.8 μg/kg in locally produced beer and 6.7 μg/kg in beers of undisclosed origin.
Stefan Ehling & Todime M Reddy, 2015 [99].	HPLC-MS	Set of nutritional ingredients derived from soy, corn and sugar beet and also in cow’s milk and human breast milk	Glyphosate, AMPA (aminomethylphosphonic acid)	Glyphosate and AMPA were quantified at concentrations of 0.105 μg/g and 0.210 μg/g, respectively, in isolated soy protein. In soy protein concentrate, glyphosate and AMPA were quantified at concentrations of 0.850 μg/g and 2.71 μg/g, respectively.
Narong Chamkasem, 2017 [100].	LC-MS-MS	Grapes	Glyphosate	At concentrations of 100, 500, and 2000 ng/g (*n* = 5), the average recovery for all analytes ranged from 87 to 111%, with a relative standard deviation of less than 17%.
Alistair K Brown & Annemieke Farenhorst, 2024 [101].	UHPLC-ESI-MS/MS	Water	Glyphosate, AMPA (aminomethylphosphonic acid)	Tap and surface water samples were analyzed at concentrations of 2 and 20 μg/L. The LOD and LOQ ranged from 0.022/0.074 to 0.11/0.36 μg/L, with precision levels of 0.46–2.2% (intraday) and 1.3–7.3% (interday). In tap water, mean pesticide concentrations in μg/L were as follows: AMPA 0.11 (0.007), glufosinate and glyphosate below the LOD. In the Red River water, AMPA was 0.56 (0.045), glufosinate below the LOQ, and glyphosate 0.40 (0.072). Glufosinate concentrations were above the LOD but below the LOQ for smaller tributaries, with a concentration of 0.2 μg/L.
Martin A. Amberger et al., 2023 [54].	LC-ESI-MS/MS	Apple, mushrooms, grapefruit, flaxseed, red lentils, and wheat	Glyphosate, AMPA (aminomethylphosphonic acid)	LODs have been established for several samples, including apple, mushroom, grapefruit, flaxseed, red lentil, and wheat. These LODs ranged from 0.09 to 0.8 µg/kg for glyphosate, from 0.04 to 1 µg/kg for AMPA, and from 0.2 to 2 µg/kg for glufosinate. Recoveries ranged from 84% to 120%, while RSD ranged from 1% to 19% for glyphosate, AMPA, and glufosinate at all fortification levels in all matrices investigated.
Selim A. Alarape et al., 2023 [102].	HPLC	Fish	Glyphosate, AMPA (aminomethylphosphonic acid)	The presence of glyphosate residues was reported in all 75 fish tissue samples.

Abbreviations: AMPA (aminomethylphosphonic acid), MRL (Maximum Residue Limit), LC-MS/MS (liquid chromatography coupled with triple quadrupole mass spectrometry), LOQ (limit of quantification), MRL (Maximum Residue Limit), HPLC-ESI-MS/MS (high-performance liquid chromatography coupled with electrospray ionization tandem mass spectrometry), LOD (limit of detection), GBHs (glyphosate-based herbicides), HQ (Hazard Quotient), ADI (Acceptable Daily Intake), ARfD (Acute Reference Dose), HPLC (high-performance liquid chromatography), HPLC-ICP-MS/MS (triple quadrupole inductively coupled plasma mass spectrometry coupled with high-performance liquid chromatography), RSD (Relative Standard Deviation), MRM (Multiple Reaction Monitoring), UHPLC-ESI-MS/MS (ultra-high-performance liquid chromatography-electrospray ionization-tandem mass spectrometry), LC-ESI-MS/MS (liquid chromatography-electrospray tandem mass spectrometry), UHPLC (ultra-high performance liquid chromatography—tandem mass spectrometry), ILIS (Labeled Internal Standards).

Several studies also investigate the presence of glyphosate and its metabolite, aminomethylphosphonic acid (AMPA), in individuals not occupationally exposed, suggesting that the pesticide source may be food and water ingestion. These findings highlight the potential for widespread population exposure to glyphosate, even in individuals not directly involved in its handling or application. This technique has also been used to identify pesticide residues in various foods. Detecting glyphosate and AMPA in foods is crucial for assessing the potential health risks associated with their consumption. Using the HPLC (high-performance liquid chromatography) technique, it is possible to identify glyphosate and AMPA residues in various food matrices, including fruits, vegetables, grains, and products of animal origin [90].

A study was initiated to evaluate the contamination of surface waters by pesticides using the HPLC technique to measure the concentrations of glyphosate and AMPA. The LOD was set at 0.0125 mg/L for glyphosate and AMPA, respectively, and the LOQ was 0.025 for glyphosate and 0.25 mg/L for AMPA. Glyphosate was found in 37.1% of water samples, with 19.3% detected and 17.7% quantified (0.35–1.65 mg/L). In 21.8% of the samples, AMPA was found: 20.2% was detected and 1.6% was quantified (0.55–0.75 mg/L). Extra samples were collected on days with precipitation more significant than 10mm, and these revealed that there was glyphosate in 65.2% of the samples, with 27.5% detected and 37.7% quantified (0.31–0.91 mg/L). AMPA was found in 33.5% of samples, 24.8% was detected, and 8.7% was quantified (0.50–1.40 mg/L). This finding makes it clear that water flow in the soil is an essential source of pesticides [89].

A study was carried out to identify the possible contamination of groundwater with pesticides using the technique UHPLC-ESI-MS/MS (ultra-high-performance liquid chromatography-electrospray ionization-tandem mass spectrometry), which identified glyphosate and AMPA. Ninety-seven samples were collected, and glyphosate was detected in 40 ± 10% of the water samples, with 41 ± 11% at values above the Environmental Quality Standard for Groundwater (EQS GW—Environment Quality Standard for groundwater). Conversely, AMPA was detected at values between <50 and 8500 ng/L in 55 ± 2% of the samples collected, of which 56 ± 14% were above the EQS GW. According to the authors, the high concentrations of the compounds in the water were not expected since, according to climate estimates, there would be no risk of leaching into groundwater [94].

A Brazilian study conducted to analyze the concentrations of pesticide residues in the soil identified glyphosate and AMPA in quantities not previously described in the literature. The analysis of the compounds present in the samples was carried out using high-performance liquid chromatography coupled with a fluorescence detector. Glyphosate and AMPA were found in all soil samples at maximum concentrations of 66.38 mg/kg and 26.03 mg/kg, respectively. Another discovery was the presence of dichlorodiphenyltrichloroethane (DDT) and its metabolites in the soil, despite the insecticide being banned in Brazil since 2009 [103].

Maria C. Fontanella and colleagues [90] successfully detected glyphosate in both white and brown rice samples using high-performance liquid chromatography coupled with inductively coupled plasma mass spectrometry (HPLC-ICP-MS). They employed a solution of methanol and a PRP-X100 anionic column, achieving average recoveries ranging from 76% to 105% across three fortification levels. A linear response was observed in all instances throughout the entire concentration spectrum.

Another study was carried out using the technique LC-MS/MS (liquid chromatography coupled with triple quadrupole mass spectrometry) to evaluate the presence of pesticide contaminants, which involved the analysis of several honey samples to verify the presence of glyphosate and AMPA. The LOQ (limit of quantification) was set at 0.04 µg/g for both compounds. A total of 38% of the samples analyzed presented glyphosate and AMPA levels above the LOQ. Six samples contained levels 4.4 times higher than the Maximum Residue Limit indicated by the European Union (0.05 µg/g). This finding highlights that the use of pesticides affects the entire ecosystem around them [91].

Since Brazil is one of the largest soy exporters in the world, a study began to evaluate pesticide residues in soy-based infant formulas. As transgenic soybeans are tolerant to the herbicide glyphosate, the product is more used in crops, increasing contamination. The samples were purchased on the local market over five years and analyzed by HPLC. Glyphosate residues ranged from 0.03 mg/kg to 1.08 mg/kg, and AMPA residues from 0.02 mg/kg to 0.17 mg/kg. This represents the inaugural scientific communication regarding glyphosate and AMPA contamination in soy-based infant formulas in Brazil. We understand the problematization of the detection of glyphosate in infant formulas due to the congruence of the facts. Study in this field is still necessary [96].

## 5. Evidence concerning Human Contamination by Glyphosate and Risk Assessment

The indiscriminate use of pesticides has led to environmental accumulations and, consequently, increases in food contamination levels. When applied to crops, pesticides widely contaminate soil, water, and air, negatively interfering with natural ecosystems and biodiversity. These products also remain in food even after washing and preparation, posing a risk to human health [104,105,106]. As a consequence of this exposure, detecting the pesticide in the population’s biological samples is possible, as mentioned in Table 3.

In the United States, it was regularly identified as the second most used crop protection product in the home and garden market sector between 2001 and 2007, with annual use of 2000 to 4000 tons [107]. Therefore, there are concerns about its excessive use’s possible environmental and health effects. The risks of carcinogenicity in humans have not yet been established. On 20 March 2015, the International Agency for Research on Cancer (IARC), linked to the World Health Organization, released a report in which glyphosate was classified as a probable carcinogen, group 2A [108]; this research brought together a working group with the participation of 17 experts from 11 countries for seven days.

Despite this classification, evidence of carcinogenicity in humans is limited, as glyphosate contamination in humans comes mainly through occupational exposure, where people who work with the herbicide application can come into direct contact with the product. Schinasi and Leon [109], coordinated a systematic review and meta-analysis regarding non-Hodgkin’s lymphoma and occupational exposure to agricultural pesticides. He found a meta-risk ratio of 1.5 (95% CI, 1.1–2.0). Contamination can also occur indirectly through the ingestion of food and water containing glyphosate residues. According to a 2017 report by the UN on food safety, pesticides pose a significant health risk to consumers who are exposed to multiple residues daily [110].

A study by Siriporn Thongprakaisang and colleagues [111] demonstrated that glyphosate, at a concentration of parts per trillion (ppt), induces the proliferation of human breast cancer cells. According to the same authors, the work demonstrated that glyphosate is “less toxic than other pesticides”, this condition does not weaken the “potential adverse health effects for humans”, as it is also related to endocrine changes. Other studies corroborate the previously cited evidence on levels of human exposure to glyphosate, including the general population and occupationally exposed workers, such as the systematic literature review carried out by Gillezeau et al. [112] and the meta-analysis conducted by Zhang et al. [113], who evaluated the potential association between high and cumulative exposure to glyphosate and the risk of developing non-Hodgkin lymphoma in humans. This study indicates that there is an evident correlation between exposure to glyphosate and the increasing threat of non-Hodgkin’s lymphoma. The IARC reinforces these findings and concludes “that there is ‘strong evidence’ that exposure to glyphosate is genotoxic through at least two devices considered to be correlated with human carcinogens, which are DNA damage and oxidative stress” [25].

Another critical point to highlight is that there is convincing evidence that glyphosate can also cause cancer in laboratory animals [108]. This assessment is based on evidence of genotoxicity for “pure” glyphosate and for formulations of this herbicide. In a study with groups of 50 male and 50 female CD-1 mice, they were offered diets containing glyphosate (purity, 99.7%) at a concentration of 0, 1000, 5000, or 30,000 ppm, ad libitum for 24 months. The group that received the highest concentration showed a consistent decrease in body weight in male and female mice compared to controls. Survival in all dose groups was similar to controls. There was a positive trend (*p* = 0.016) in the incidence of renal tubule adenoma in dosed male mice [108]. Another study described to the Joint FAO/WHO Meeting on Pesticide Residues (JMPR), with groups of 50 male and 50 female CD-1 mice that received pure glyphosate (98.6%), also showed that there was a tendency towards an increase in incidence of hemangiosarcoma in male mice (8%) and a trend toward increased incidence of histiocytosis sarcoma in hemopoietic tissue (not statistically significant for men or women) [114].

Detecting glyphosate and its metabolites in human fluids of not occupationally exposed individuals strongly suggests continuous contamination of people linked to food and water ingestion. Even given the crucial role of vegetables and fruits in nourishing and preventing chronic diseases, consuming contaminated food can have critical consequences. Conventional food cultivation, due to the extensive use of glyphosate, constitutes a chronic risk for the development of cancer, given the potential and frequent carcinogenic nature present, often exceeding maximum residue limits [115]. A dietary cancer risk estimative has been discussed concerning pesticides as food contaminants, based on these MRLs, as recently addressed by Valentim and Cols (2023) [115].

A study was carried out to evaluate data on food consumption and pesticide exposure using the LC-MS/MS technique, and glyphosate and AMPA were identified. About 8.3% of samples exhibited quantifiable concentrations (>0.2 µg/L) of glyphosate and/or AMPA in urine, with maximum concentrations being 1.36 and 1.53 µg/L, respectively. The group of participants with LOD ≥ 0.05 µg/L and ≥0.09 µg/L for glyphosate and AMPA, respectively, exhibited concentrations of GLY in a range from 0.05 to 1.36 µg/L and of AMPA in a range from 0.09 to 1.53 µg/L. There was a significant correlation between the consumption of legumes and urinary excretion of glyphosate (*p* < 0.0001) and between the consumption of mushrooms and urinary excretion of AMPA (*p* = 0.0102). The sum of glyphosate and AMPA excretion is also associated with the consumption of legumes (*p* = 0.0003) [116].

A study evaluating glyphosate levels and their association with food consumption [117] identified glyphosate residues in 89.9% of urine samples and AMPA residues in 67.2% of urine samples. These samples were analyzed using LC-MS/MS in a group of postmenopausal women. The LOD and LOQ for glyphosate were 0.014 and 0.041 ng/mL, respectively. The LOD and LOQ for AMPA were 0.013 and 0.040 ng/mL, respectively. Median concentrations were 0.10 and 0.04 ng/mL for glyphosate and AMPA, respectively, with a maximum of 3.01 ng/mL for glyphosate and 1.51 ng/mL for AMPA. Grain consumption was significantly associated with higher urinary glyphosate levels, even among women who reported consuming organic grains frequently or always (*p* = 0.002).

**Table 3:** Studies concerning glyphosate detection in human samples.

Autors	Analytical Method	Sample	Residue	Considerations
Feng Zhang et al., 2020 [37].	GC-MS	Urine	Glyphosate, AMPA (aminomethylphosphonic acid)	Urinary glyphosate concentrations ranged from <0.020 to 17.202 mg/L, and AMPA concentrations ranged from <0.010 to 2.730 mg/L.
Parvez et al., 2018 [118].	LC-MS/MS	Urine	Glyphosate	A total of 93% of women had urine glyphosate concentrations exceeding the LOD of 0.1 µg/L, with a mean concentration of 3.40 µg/L.
Eick Sierra-Diaz et al., 2019 [119].	HPLC-MS/MS	Urine	Glyphosate	Glyphosate was identified in 73% and 100% of individuals tested from two distinct communities, with average urine glyphosate concentrations of 0.36 and 0.61 µg/L, respectively.
Alison Connolly et al., 2018 [120].	LC-MS/MS	Urine	Glyphosate	The median and maximum concentrations of glyphosate found in the ten samples were 0.87 and 1.35 µg/L, respectively.
LeonardoTrasande et al., 2020 [121].	LC-MS/MS	Urine	Glyphosate	Glyphosate was detectable in 30%, 12.5%, and 7.6% of infants/children in the <30 days, 10–19 months, and 3–8 years age categories, respectively. The average detectable concentration of glyphosate in urine was 0.278 µg/L, with concentrations ranging from 0.105 to 2.125 µg/L.
Anja Stajnko et al., 2020 [122].	GC-MS/MS	Urine	Glyphosate, AMPA (aminomethylphosphonic acid)	Glyphosate and AMPA were found in 27% and 50% of urine samples collected during the first sampling period, respectively. In the second sampling period, they were detected in 22% and 56% of the samples, respectively.
Sebastian T Soukup et al., 2020 [116].	LC-MS/MS	Urine	Glyphosate, AMPA (aminomethylphosphonic acid)	A total of 8.3% of participants (*n* = 25) had quantifiable concentrations (>0.2 µg/L) of glyphosate and/or AMPA in their urine. Glyphosate was not detected (<0.05 µg/L) in 66.5% of the samples, and AMPA was not detected (<0.09 µg/L) in the same percentage.
Pablo Ruiz et al., 2021 [123].	LC-MS/MS	Urine	Glyphosate, AMPA (aminomethylphosphonic acid)	The detection frequencies (DFs) generated rates of 54% for glyphosate and 60% for AMPA. The GMs of the EDIs were determined to be 0.31 and 0.37 μg/kg body weight/day for glyphosate and AMPA, respectively.
Melissa J. Perry et al., 2019 [34].	LC-MS/MS	Urine	Glyphosate	The average level of glyphosate detected was 4.04 μg/kg (equivalent to 4.04 ppb) in the seven positive samples, ranging from 1.3 to 12.0 μg/kg.
Paulo Nova et al., 2020 [124].	GC-MS and HPLC-MS	Urine	Glyphosate, AMPA (aminomethylphosphonic acid)	During the initial testing phase, 28% and 50% exhibited detectable levels of glyphosate and AMPA, respectively, with median values of 0.25 and 0.16 μg/L. In the second round, 73% and 97% revealed detectable levels of glyphosate and AMPA, respectively, with median values of 0.13 and 0.10 μg/L.
Hiroshi Nomura et al., 2022 [125].	LC-MS/MS	Urine	Glyphosate	Glyphosate was detectable in 41% of the 234 children studied. The 75th percentile and maximum urine glyphosate concentrations were recorded at 0.20 and 1.33 μg/L, respectively.
Robin Mesnage et al., 2022 [126].	LC-MS/MS	Urine and feces	Glyphosate	Glyphosate was detected in 53% of urine samples, but below the LOQ (<0.1 μg/L) in 10 cases (8%).
Ana P. Balderrama-Carmona et al., 2020 [127].	HPLC	Urine	AMPA	Urine samples (*n* = 30) revealed concentrations of up to 10.25 μg/L of picloram and 2.23 μg/L of AMPA, with no reports of positive samples for glyphosate.
Raquel Lúcia et al., 2023 [117].	LC-MS/MS	Urine	Glyphosate, AMPA (aminomethylphosphonic acid)	Glyphosate was detected in 89.9% of urine samples, while AMPA was found in 67.2%.
Felipe Lozano-Kasten et al., 2021 [128].	HPLC-MS	Urine	Glyphosate	All samples yielded positive results for glyphosate levels. Glyphosate is pervasive among children of all ages within the community, even in cases where they have not experienced direct exposure to it.
Roy R. Gerona et al., 2022 [129].	HPLC-MS	Urine	Glyphosate	Glyphosate was detected in 99% of pregnant women. High maternal levels during the first trimester correlated with lower body weight percentiles and increased risk of intensive care unit admission.
Garth Campbell et al., 2022 [38].	LC-MS/MS	Urine	Glyphosate, AMPA (aminomethylphosphonic acid)	Glyphosate has been found above the LOD (ranging from 0.20 to 1.25 μg/L) in 8% of the Australian population. Glyphosate (ranging from 0.85 to 153 μg/L) and AMPA (ranging from 0.50 to 3.35 μg/L) were detected in 96% and 33% of farmers, respectively.
Imane Berni et al., 2023 [39].	LC-MS/MS	Urine	Glyphosate, AMPA (aminomethylphosphonic acid)	Glyphosate and AMPA were detected in 73% and 75% of urine samples, respectively.

Abbreviations: AMPA (aminomethylphosphonic acid), MRL (Maximum Residue Limit), LC-MS/MS (liquid chromatography coupled with triple quadrupole mass spectrometry), LOQ (limit of quantification), MRL (Maximum Residue Limit), HPLC-ESI-MS/MS (high-performance liquid chromatography coupled with electrospray ionization tandem mass spectrometry), LOD (level minimum detection), GBHs (glyphosate-based herbicides), HQ (Hazard Quotient), ADI (Acceptable Daily Intake), ARfD (Acute Reference Dose), HPLC (high-performance liquid chromatography), HPLC-ICP-MS/MS (triple quadrupole inductively coupled plasma mass spectrometry coupled with high-performance liquid chromatography), GLY (glyphosate), RSD (Relative Standard Deviation), MRM (Multiple Reaction Monitoring), UHPLC-ESI-MS/MS (ultra-high-performance liquid chromatography-electrospray ionization-tandem mass spectrometry), LC-ESI-MS/MS (liquid chromatography-electrospray tandem mass spectrometry), UHPLC-MS/MS (ultra-high performance liquid chromatography—tandem mass spectrometry), ILIS (Labeled Internal Standards), GC-MS (gas chromatography-mass spectrometry), TWA (Time-Weighted Average), DFs (detection frequencies), EDIs (Estimated Daily Intakes), HI (Hazard Index), EFSA (European Food Safety Authority).

Urinary glyphosate-AMPA residues were investigated in workers involved in herbicide production by using GC-MS/MS [37]. The detection rates were 86.6% and 81.3%, respectively. The limit of determination (LOD) was 0.02 mg/m³. Workers involved in centrifugation, crystallization, drying, packaging, and feeding were exposed to glyphosate, with the packaging sector being the most affected. There was a significant difference in the urinary concentration of the compounds between the different jobs (*p* < 0.05).

Another study, carried out to assess exposure to glyphosate in pregnancy and the duration of pregnancy using the LC-MS/MS technique, reported that 93% of pregnant women had detectable urinary levels of glyphosate, with an average concentration of 3.40 ng/mL. Higher levels of glyphosate were observed in women living in rural areas who consumed more caffeine. The minimum and maximum glyphosate urinary concentrations among pregnant women were 0.5 ng/mL and 7.20 ng/mL, respectively. Higher levels of glyphosate in urine were significantly correlated (*p* = 0.02) with shorter gestational periods [118].

A study evaluated prenatal exposure to glyphosate and birth outcomes to establish the levels of glyphosate in the urine of a cohort of high-risk pregnant women. In the first trimester of pregnancy, random urine samples were collected and analyzed by LC-MS/MS. The LOQ and LOD limits were 0.5 and 0.1 ng/mL, respectively. Glyphosate levels in urine ranged from 0.10 to 6.89 ng/mL. Newborn body weight percentiles were negatively related to glyphosate in the mother’s urine (*p* = 0.023) [129].

Biomarkers of glyphosate exposure and their possible associations with kidney function in children were also investigated. Glyphosate was detected in 11.1% of participants, with a higher frequency in the neonatal population. Additionally, it was detectable in 30% of children aged less than 30 days, in 12.5% aged between 10 and 19 months, and in 7.6% aged between 3 and 8 years. The average detectable concentration of glyphosate using the LC-MS/MS in urine was 0.278 µg/L, and concentrations ranged from 0.105 to 2.125 µg/L. However, there was no association between glyphosate levels and biomarkers of kidney damage [121].

One study aimed to estimate the exposure to glyphosate and AMPA among children and adolescents living in agricultural areas in Slovenia. Urine samples were collected from 149 children (aged from 7 to 10 years) and 97 adolescents (aged from 12 to 15 years) and analyzed using GC-MS/MS. The Limit of Quantification (LOQ) and Limit of Detection (LOD) were 0.1 µg/L. The study did not find a significant association between exposure and the reported use of glyphosate or herbicides near the participants’ homes or in the vicinity of agriculture, revealing the potential for widespread exposure of the population to pesticides through environmental contamination [122].

Glyphosate was analyzed in another study using frozen urine samples collected between 1997 and 1998 among a population of farmers in the USA. LC-MS/MS analyzed the samples and showed an LOD of 0.4 μg/kg (0.4 ppb) for glyphosate and 1 μg/kg (1 ppb) for AMPA. Among the farmers who reported using pesticides, 39% showed detectable levels of glyphosate, with values ranging from 1.3 to 12.0 μg/kg and with an average concentration of 4.04 μg/kg (4.04 ppb). This finding provides important information on the bioavailability of glyphosate even after freezing [34].

The exponential increase in glyphosate consumption over the years has led the scientific community to question the possible toxicity of this herbicide and its possible effects on human health [24]. Consequently, scientific studies published on the impact of this herbicide and its metabolites have shown a significant increase in humans and the environment [10].

Several researchers have evaluated the impact of glyphosate and their results have indicated that, even at low concentrations of the herbicide, its commercial formulations cause numerous pathologies [130,131,132]. Glyphosate has been identified as a contributing factor in the development of the autism and obesity epidemics in the United States, as well as different diseases such as infertility, Parkinson’s, depression, Alzheimer’s, and cancer [133]. Especially after the reclassification of glyphosate in 2015 by the IARC, the product was alerted for human health due to its probable carcinogenicity [44]. There are controversies between the private sector and the scientific community about glyphosate, especially food safety and its health causes [24].

The toxicity of glyphosate’s primary metabolite, aminomethylphosphonic acid (AMPA), is lower or equivalent to that of the original compound [134]. In this context, three pillars emerged as the problems to be investigated in human health. These are toxicological parameters, acute toxicity, and chronic toxicity, as shown in Table 4.

According to the European Food Safety Authority (EFSA) [135], in vivo studies have defined the following toxicological parameters: Acceptable Daily Intake Level (0.5 mg/kg body weight per day), Acceptable Operator Exposure Level (AOEL) (0.1 mg/kg body weight per day), No Observable Adverse Effect Level (NOAEL) (100 mg/kg body weight per day), and Acute Reference Dose (ARfD) (0.5 mg/kg body weight per day).

In terms of acute toxicity, it is classified as a category IV non-toxic substance [136]. According to data from the European Chemicals Agency (ECHA), exposure to glyphosate has caused severe eye irritation and damage. In addition, in humans, the toxicity of this herbicide on contact through accidental or intentional ingestion causes liver, kidney, gastrointestinal, and lung disorders and weight loss [18,137].

**Table 4:** Chronic toxicity, body targets, and consequences of glyphosate exposure in humans.

Chronic Toxicity	Body Targets	Consequences of Exposure to Glyphosate	References
Target organ toxicity	Gastrointestinal, Heart, Liver, Kidneys	Celiac disease, Electrocardiogram abnormalities and arrhythmias, Oxidative stress Non-alcoholic fatty liver disease, steatohepatitis and liver dysfunction, Chronic kidney disease	Lola Rueda-Ruzafa et al. 2019 [138]. Steeve Gress et al. 2015 [139]. Ryan Brunetti et al. 2020 [140]. Robin Mesnage et al. 2015 [130]. Robin Mesnage et al. 2017 [141]. Hui Gao et al. 2019 [142].
Cytotoxicity	Human red blood cells	Morphological changes.	Islam Md. Meftaul et al. 2020 [132].
Neurotoxicity	Human neuronal cells.	The dysfunction of acetylcholinesterase disrupts the regulation of nerve impulse transmission, thereby contributing to the development of neurological disorders.	Van Bruggen A.H.C et al. 2017 [25].
Genotoxicity	Deoxyribonucleic acid (DNA), Human leukocytes.	Mammalian chromosomes are harmed, leading to epigenetic changes, including DNA methylation and the promotion of histone modification.	Kathryn Z Guyton et al. 2015 [143]. Marta Kwiatkowska et al. 2017 [144]. María F. Rossetti et al. 2021 [145].
Teratogenic effects	Ferns	Malformations	M. Antoniou et al. 2012 [146].
Endocrine disruption	Hormonal axis	Endocrine system disorders	Robin Mesnage et al. 2015 [130]. EFSA et al. 2016 [135].

Abbreviations: Deoxyribonucleic acid (DNA).

Exposure to glyphosate can occur mainly through oral, pulmonary (respiratory), or dermal contact [135]. Contact via the dermal route is the one that receives the most reports from workers due to the absorption of the element in the body [147]. It can be detected in the vital organs (liver, colon, small intestine, kidneys) due to accumulation and excretion of the element. In most cases, it can occur in the feces, 90%, and in the urine up to 48 h after exposure [135].

The evidence in the literature indicates a significant impact on human health when individuals are exposed to glyphosate, especially in Brazil, where exposure is a high continuous flow. The impact reflects direct and indirect handling, with only the quantity and concentration of these products varying [148,149]. It should be noted that children are more vulnerable to glyphosate than adults and older adults due to their behavioral and physiological differences [115].

## 6. Conclusions

There has been a significant global increase in glyphosate use over the last few decades, leading to undeniable food and human contamination. Despite numerous studies highlighting the potentially harmful effects of glyphosate, there is still a contradiction among global organizations and regulatory bodies regarding this herbicide. The Risk Assessment Committee (RAC) of the European Chemicals Agency [150] concluded that the available scientific evidence did not meet the criteria for classifying glyphosate as carcinogenic, mutagenic, or toxic for reproduction. However, it is crucial to adhere to proper usage guidelines and be aware of local regulations concerning glyphosate. The fundamentals of pesticide release and use are being challenged due to the global identification of present and future dangers, as science progresses. More reliable analytical methods beyond LC and GC-MS need to be developed. Unfortunately, access to such technologies is often restricted due to their high costs, particularly in the most contaminated nations, such as developing countries. Independent research, published and validated by experts, along with solid evidence demonstrating the risks associated with food contamination by glyphosate, requires more attention to improve public safety.

## Figures and Tables

**Table 1:** Glyphosate chemical properties.

Common Name	Glyphosate—Ammonium Salt (Glyphosate-Ammonium)
IUPAC nomenclature	2-(phosphonomethylamino) acetic acid
Chemical name	Ammonium N-[(hydroxyphosphinato) methyl]glycine
CAS No.	114370-14-8
Synonymy	CP 67573 Glyphosate
Chemical Group	Substituted Glycine
Class of use	Herbicide
Molar mass	169.05 g/mol
Molecular formula	C_3_H_7_NO_5_P. × NH_3_
Relevant impurities	N-nitrosoglyphosate: 0.001 g/kg formaldehyde: 1.3 g/kg
Physical state, appearance, color, and odor	Crystalline solid, odorless, and colorless.
Fusion point	It cannot be determined up to a temperature of 250 °C using the capillary method. The product decomposes before melting (~235 °C).
Hydrolysis (1/2 life time and Conditions)	>30 days (pH 5; 7; 9; 25 °C)
Photolysis (1/2 life time and Conditions)	01 days (soil; pH 6.1; 22 ± 2 °C)
Surface tension of solutions (1/2 life time and conditions)	73.0 nN/m (20 °C)
Density	1655 g/mL (20 °C)
Thermal and air stability	Stable under heating condition of 50 ± 5 °C. Stable at room temperature for 28 days.
Volatility	Henry’s constant = 2.08 × 10^−12^ atm × m^3^ × mol^−1^
Solubility in water	252.9 mg/mL (pH 3,6; 20 °C) >353 mg/mL (pH 7; 20 °C) >340 mg/mL (pH 9; 20 °C)
Acetone Solubility	0.078 g/L (20 °C)
Ethyl acetate solubility	0.012 g/L (20 °C)
Dissociation constant in aqueous medium	pka 1 = 2.72 (25 °C) pka 2 = 5.63 (25 °C) pka 3 = 10.2 (25 °C)
Complex formation constant with metals in aqueous medium	Low capacity for complex formation with copper, cadmium, and lead.

Source: National Institutes of Health (NIH)—PubChem [45].

## Data Availability

The original contributions presented in the study are included in the article, further queries can be directed to the corresponding author.

## Supplementary Materials

The following supporting information can be downloaded at: https://www.mdpi.com/article/10.3390/foods13111697/s1, Table S1: Glyphosate detection in food according to the PARA (Food Pesticide Residue Analysis Program) Brazilian report, 2018, Table S2: Glyphosate detection in food according to the EFSA (European Food Safety Authority) report, 2018–2020, Table S3: Glyphosate detection in food according to the European Union Report, 2019, Table S4: Glyphosate detection in food according to the FDA (Food and Drug Administration) report, 2018–2019, Table S5: Glyphosate detection in food according to the CFIA (Canadian Food Inspection Agency) report, 2015–2016.
