# Supplementary material for: Glyphosate as a Food Contaminant: Main Sources, Detection Levels, and Implications for Human and Public Health
Source: Foods. 2024 May 28;13(11):1697. doi: 10.3390/foods13111697 (PMC11171990; doi:10.3390/foods13111697)
Supplement: Supplementary file 1 [file foods-13-01697-s001.zip › foods-2970732-supplementary.pdf]

**Supplemnetary Table S1:** Glyphosate detection in food according to the PARA (Food Pesticide Residue Analysis Program) Brazilian report, 2018.

| Food | MRL (mg/kg) | Number of positive analyses |
|---|---|---|
| Rice | 0.2 | 329 |
| Grape | 0.2 | 319 |

**MRL (Maximum Residue Limit)**

**Supplemnetary Table S2:** Glyphosate detection in food according to the EFSA (European Food Safety Authority) report, 2018–2020.

| Food | Level detected | Number of analysed samples | Number of positive samples | MRL (mg/kg) |
|---|---|---|---|---|
| Barley | 0.01 | 152 | 18 | 20 |
| Buckwheat | 0.01 | 152 | 16 | 0.1 |
| Lentil | 0.01 | 238 | 82 | 10 |
| Oat | 0.002 | 113 | 2 | 20 |
| Honey | 0.01 | 563 | 51 | 0.5 |
| Rye | 0.002 | 759 | 31 | 10 |
| Wheat | 0.002 | 1234 | 50 | 10 |

**MRL (Maximum Residue Limit)**

**Supplementary Table S3:** Glyphosate detection in food according to the European Union Report, 2019.

| Food | % of samples under MRL | % of samples over MRL |
|---|---|---|
| Bean | 11.1 | 2.8 |
| Millet | 7.7 | 7.7 |
| Honey | 6 | 0.8 |
| Buchwheat | 1.9 | 3.7 |
| Peach | 1.6 | 0.1 |
| Spinach | 0.8 | 0.2 |
| Cabbage | 0.3 | 0.2 |

**MRL (Maximum Residue Limit)**

**Supplementary Table S4:** Glyphosate detection in food according to the FDA (Food and Drug Administration) report, 2018-2019.

| Food | Level detected | Number of analyzed samples | Number of positive samples | MRL (mg/kg) |
|---|---|---|---|---|
| Soy | 0.01 | 337 | 206 | 20 |
| Corn | 0.01 | 313 | 180 | 5 |
| Milk | 0.01 | 121 | 0 | - |
| Eggs | 0.01 | 108 | 0 | 0.05 |

**MRL (Maximum Residue Limit)**

**Supplementary Table S5:** Glyphosate detection in food according to the CFIA (Canadian Food Inspection Agency) report, 2015-2016.

| Food | Number os samples | % of positive samples | % of samples over MRL |
|---|---|---|---|
| Fresh fruits and vegetables | 317 | 7.3 | 0 |
| Processed fruits and vegetables | 165 | 12.1 | 0 |
| Grain products | 869 | 36.6 | 3.9 |
| Beverages | 496 | 16.3 | 0.2 |
| Products based on beans, lentils and peas | 869 | 47.4 | 0.6 |
| Soy-based products | 263 | 11 | 0 |

**MRL (Maximum Residue Limit)**
